# Dual Neonate Vaccine Platform against HIV-1 and *M. tuberculosis*

**DOI:** 10.1371/journal.pone.0020067

**Published:** 2011-05-13

**Authors:** Richard Hopkins, Anne Bridgeman, Joan Joseph, Sarah C. Gilbert, Helen McShane, Tomáš Hanke

**Affiliations:** 1 MRC Human Immunology Unit, Weatherall Institute of Molecular Medicine, University of Oxford, Oxford, United Kingdom; 2 AIDS Research Unit, Hospital Clínic/IDIBAPS-HIVACAT, School of Medicine, University of Barcelona, Barcelona, Spain; 3 The Jenner Institute, University of Oxford, Oxford, United Kingdom; Tsinghua University, China

## Abstract

Acquired immunodeficiency syndrome and tuberculosis (TB) are two of the world's most devastating diseases. The first vaccine the majority of infants born in Africa receive is *Mycobacterium bovis* bacillus Calmette-Guérin (BCG) as a prevention against TB. BCG protects against disseminated disease in the first 10 years of life, but provides a variable protection against pulmonary TB and enhancing boost delivered by recombinant modified vaccinia virus Ankara (rMVA) expressing antigen 85A (Ag85A) of *M. tuberculosis* is currently in phase IIb evaluation in African neonates. If the newborn's mother is positive for human immunodeficiency virus type 1 (HIV-1), the baby is at high risk of acquiring HIV-1 through breastfeeding. We suggested that a vaccination consisting of recombinant BCG expressing HIV-1 immunogen administered at birth followed by a boost with rMVA sharing the same immunogen could serve as a strategy for prevention of mother-to-child transmission of HIV-1 and rMVA expressing an African HIV-1-derived immunogen HIVA is currently in phase I trials in African neonates. Here, we aim to develop a dual neonate vaccine platform against HIV-1 and TB consisting of BCG.HIVA administered at birth followed by a boost with MVA.HIVA.85A. Thus, mMVA.HIVA.85A and sMVA.HIVA.85A vaccines were constructed, in which the transgene transcription is driven by either modified H5 or short synthetic promoters, respectively, and tested for immunogenicity alone and in combination with BCG.HIVA^222^. mMVA.HIVA.85A was produced markerless and thus suitable for clinical manufacture. While sMVA.HIVA.85A expressed higher levels of the immunogens, it was less immunogenic than mMVA.HIVA.85A in BALB/c mice. A BCG.HIVA^222^–mMVA.HIVA.85A prime-boost regimen induced robust T cell responses to both HIV-1 and *M. tuberculosis*. Therefore, proof-of-principle for a dual anti-HIV-1/*M. tuberculosis* infant vaccine platform is established. Induction of immune responses against these pathogens soon after birth is highly desirable and may provide a basis for lifetime protection maintained by boosts later in life.

## Introduction

Despite great efforts in distributing anti-retroviral therapy (ART) to infected mothers in resource-poor countries, universal accessibility to ART remains challenging [1]. The best solution to preventing mother-to-child transmission of human immunodeficiency virus type 1 (HIV-1) via breast-feeding, which also does not require a daily compliance, is development of an effective infant vaccine [2]. Because *Mycobacterium bovis* bacillus Calmette-Guérin (BCG) is the first vaccine of the Expanded Programme for Immunization (EPI) and technologies are now available to genetically manipulate BCG [3]–[7], we proposed to use recombinant BCG (rBCG) vaccine expressing an HIV-1-derived transgene for priming of both tuberculosis (TB)- and HIV-1-specific immune responses at birth [8]. Induced HIV-1 responses can be boosted later by a heterologous vector such as modified vaccinia virus Ankara (MVA) delivering the same HIV-1-derived transgene.

The main goal in preventing HIV-1 infection is development of a vaccine eliciting broadly neutralizing antibodies (bNAb). However, even if such a vaccine can be made [9], it will be hard to stop some virus infection occurring e.g. through cell-cell transmission and thus control of infection will require T cell-mediated immune responses. A vaccine inducing strong long-lasting T cell memory alone without bNAbs is likely to have an impact on the HIV-1 transmission.

As the first step towards a vaccine against breast milk transmission of HIV-1, we engineered BCG.HIVA^222^ vectored by a lysine auxotroph of the Pasteur strain of BCG and delivering chimaeric protein designated HIVA [8]. HIVA is the first clinically tested T cell immunogen based on consensus African HIV-1 clade A and comprizes Gag p24-p17 and a string of CD8^+^ T cell epitopes [10], [11]. In BALB/c mice, BCG.HIVA^222^ induced durable high-quality HIV-1-specific CD4^+^ and CD8^+^ T cell responses. Furthermore, when used in a heterologous prime-boost regimen, protection against surrogate virus challenge through the HIV-1-specific responses was achieved and BCG.HIVA^222^ alone protected against aerosol challenge with *M. tuberculosis* [8].

MVA is currently one of the leading candidates for development of subunit vaccines against globally important diseases. As a capacity building and the first stage towards a more complex regimen, the safety and immunogenicity of MVA.HIVA vaccine alone is under evaluation in two phase I infant vaccine clinical trials in sub-Saharan Africa [12]. The vaccine is administered to 20-week-old babies born to healthy either HIV-1-negative or positive mothers. BCG confers consistent and reliable protection against disseminated disease, but the protection against pulmonary disease is much more variable, and typically lower in tropical climates [13], [14]. MVA expressing antigen 85A (Ag85A) of *M. tuberculosis* was developed and shown to boost strongly BCG-primed and naturally acquired anti-mycobacterial immunity in humans [15]–[19] and is currently in a proof-of-concept phase IIb clinical trial evaluating the safety, immunogenicity and prevention of TB in infants primed with BCG. In the present work, we describe construction of MVA.HIVA.85A, a dual vaccine, which is designed to boost both *M. tuberculosis*- and HIV-1-specific immune responses primed by BCG.HIVA^222^.

## Results

### Construction of mMVA.HIVA.85A and sMVA.HIVA.85A

**Figure 1:**
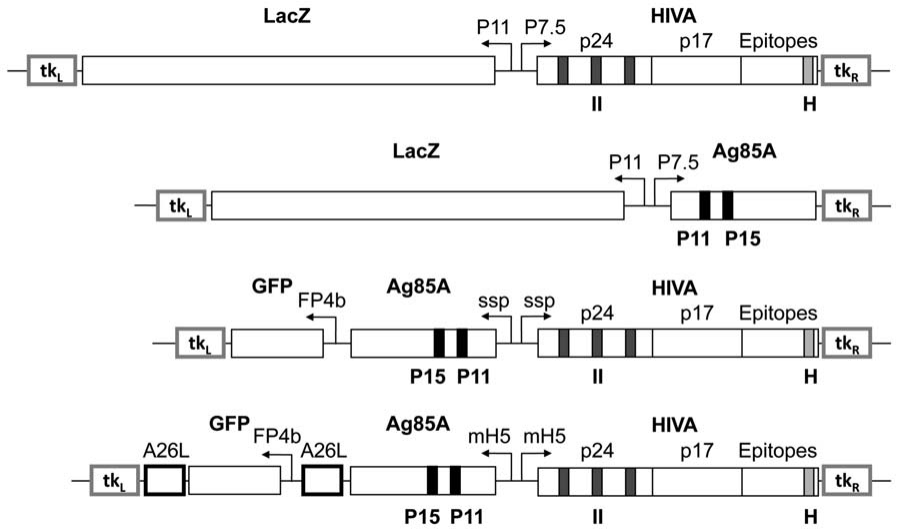
Schematic representation of inserted DNA fragments. Expression cassettes containing genes coding for immunogens HIVA and Ag85 using either β-galactosidase or GFP markers were inserted into the tk locus of the MVA genome by homologous recombination directed by the left and right tk flanking regions (tk_L_ and tk_R_). Transcription of the two immunogens was controlled by either P7.5, modified H5 (mH5) or short synthetic (ssp) promoters. In the mH5 version, the GFP gene is flanked by two homologous repeats (A26L), which facilitate removal of the selection marker. Proteins/epitopes are shown in bold, while promoters are indicated using a regular font style.

Novel experimental vaccines against pulmonary TB and breast milk HIV-1 are under development. Because protection against both of these major killers is required from birth and the first four months of life are already very busy with the scheduled EPI vaccinations, we thought it was advantageous to combine the anti-TB and anti-HIV-1 vaccine strategies into a single dual vaccine regimen consisting of priming with rBCG expressing an HIV-1-derived immunogen and a single-vaccine-construct TB/HIV-1 boost. Construction of priming BCG.HIVA was reported previously [8], [20] and individual MVA.HIVA and MVA.85A vaccines have been extensively clinically tested [10], [19] and are currently in clinical studies in neonates (HMcS and TH, unpublished). The two rMVAs drive transcription of the transgenes from early/late vaccinia virus promoter P7.5 and employ β-galactosidase gene as a marker facilitating identification of homologous recombinants (Fig. 1). However, both of these functional elements are relatively large in size, and yet smaller modified H5 (mH5) and short synthetic (ssp) promoters were reported previously for other immunogens to support higher protein expression levels compared to the P7.5 promoter [21], [22]. Furthermore, markerless rMVA is the desirable form should this vaccine become a licensed product. Therefore, mMVA.HIVA.85A and sMVA.HIVA.85A vaccines driving expression of the two immunogens from the mH5 or ssp promoters, respectively, were constructed (Fig. 1) and characterized with the aim to progress the best construct to further vaccine development. In both instances, green fluorescent protein (GFP) expression served as a selection marker for recombinant identification and for the mMVA.HIVA.85A vaccine, the marker gene was flanked by two direct 200-nucleotide repeat sequences derived from the *A26L* gene to facilitate excision of the marker via *in cis* homologous recombination resulting in a markerless rMVA.

### Short synthetic promoter supports the highest transgene expression

**Figure 2:**
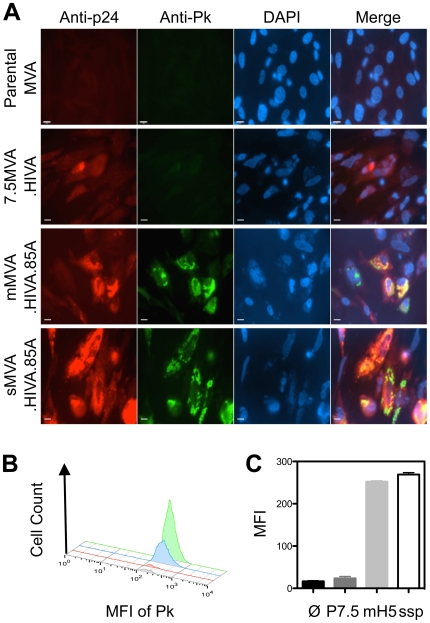
Protein expression from recombinant MVAs in CEF cells. CEF cells were infected with indicated viruses at MOI 1 for 16 h. (**A**) Cell were stained either for Gag p24 or Pk tag (on HIVA and Ag85A) expression as shown above and analyzed under fluorescent microscope. Also, the relative levels of protein expression were assessed using flow cytometry and shown either as a (**B**) histogram for parental MVA (black), 7.5MVA.HIVA (red), mMVA.HIVA.85A (blue) or sMVA.HIVA.85A (green) viruses or (**C**) expressed as mean fluorescent intensity (MFI) ± SD (**C**). The figure shows representative data of three independent experiments.

The mMVA.HIVA.85A and sMVA.HIVA.85A vaccines were first characterized as for the levels of the two transgene product expression. Both HIVA and Ag85A proteins contain a C-terminal epitope Pk recognized by monoclonal antibody SV5-P-k [23], which was attached to facilitate the immunogen detection. In addition, a mAb against p24 was employed to detect specifically the HIVA protein. Thus, monolayers of CEF cells were infected at MOI 1 with either the 7.5MVA.HIVA, mMVA.HIVA.85A or sMVA.HIVA.85A vaccines and the expression of the transgene products using the anti-Pk and p24 mAbs were readily detectable for the ssp and mH5 promoters, while for the P7.5 promoter, the immunofluorescence signal was fainter for anti-p24 mAb and almost undetectable for the Pk tag (Fig. 2A); this is similar to our previous experience [24]. To obtain more quantitative expression data, CEF cells were infected with empty parental or rMVAs and subjected to analysis by flow cytometry. The number and median fluorescent intensity (MFI) of cells expressing the Pk-epitope confirmed superior transgene expression from the sMVA.HIVA.85A ssp promoter (Fig. 2B and C).

### Modified H5 promoter provides the most immunogenic vaccine

**Figure 3:**
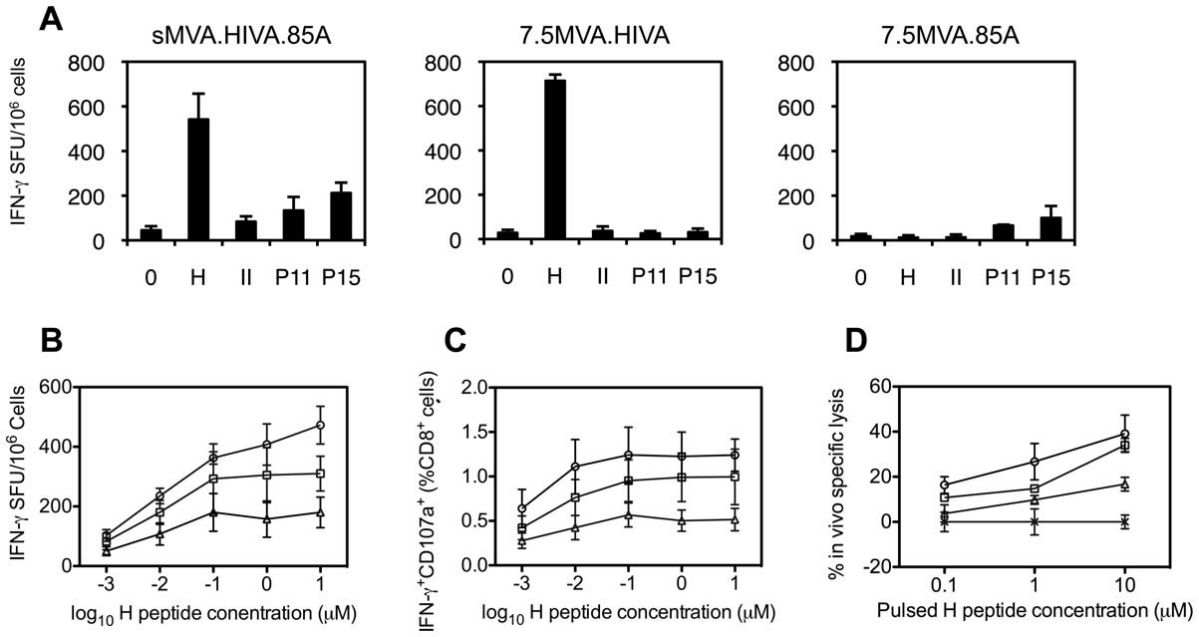
Induction of HIV-1- and *M. tuberculosis*-specific T cell responses. (**A**) Groups of 4 BALB/c mice were immunized with 10^7^ PFU i.m. of either 7.5MVA.HIVA, 7.5MVA.85A or sMVA.HIVA.85A, sacrificed 2 weeks later and splenocytes from individual mice were analyzed for IFN-γ production following peptide stimulation. Data are represented as means ± SD. (**B–C**) Groups of 4 BALB/c mice were immunized i.m. with 10^5^ PFU of either 7.5MVA.HIVA (triangles), mMVA.HIVA.85A (circles) or sMVA.HIVA.85A (squares) vaccines. Two weeks later, mice were sacrificed and the ability of splenocytes from individual animals to respond to increasing amounts of peptide H was assessed in an IFN-γ ELISPOT (**B**), ICS (**C**) and *in vivo* killing (**D**) assays. Mean ± SD are shown. Only stimulation with 1 µM H peptide in an IFN-γ ELISPOT assay provided a statistically significant difference (*p* = 0.04) between frequencies induced using the mH5 and ssp promoters.

To assess the dual vaccine immunogenicity, induction of MHC class I- and II-restricted T cell responses to the HIVA and Ag85A immunogens was compared between the dual sMVA.HIVA.85A and single 7.5MVA.HIVA and 7.5MVA.85A vaccines at the 10^7^ PFU dose delivered i.m., and found similar. For the sMVA.HIVA,85A vaccine, specific CD8^+^ T cell frequencies were dominated by responses against the H epitope, and responses to the other tested known MHC class I and II epitopes were clearly detectable (Fig. 3 A). Thus, the dual vaccine can substitute for the two single-immunogen constructs inducing both CD4^+^ and CD8^+^ T cell responses against both pathogens.

Next, CD8^+^ T cell immunogenicity between the P7.5, mH5 and ssp promoters was compared to guide the decision which construct is to be taken for further development. Groups of BALB/c mice were administered with 10^6^ PFU of rMVA i.m. and their HIVA-specific responses were assessed in a number of T cell assays 2 weeks later. Isolated splenocytes from individual mice were stimulated *in vitro* with increasing concentrations of the H peptide ranging from 0.001 µM to 10 µM and the responding cells were enumerated in an IFN-γ ELISPOT assay. The results indicated that mMVA.HIVA.85A was the most immunogenic vaccine followed by sMVA.HIVA.85A, and then 7.5MVA.HIVA, although only at 1 µM H peptide, a statistically significant difference between the mH5 and ssp promoters was detected (Fig. 3 B). Similar relative H-specific CD8^+^ T cell induction was detected using polychromatic flow cytometry confirming that the highest frequencies of IFN-γ-producing and degranulating cells were stimulated by the mMVA.HIVA.85A vaccine (Fig. 3 C). In both assays, the responses reached a plateau at 0.1 µM peptide H. Next, the immunogenicity of the 7.5MVA.HIVA, mMVA.HVA.85A and sMVA.HIVA.85A were compared in an *in vivo* killing assay, in which the target cells were pulsed with increasing concentrations of peptide H, labeled differentially with increasing concentrations of CFSE, mixed, transferred back into immunized mice and re-isolated after 5 h. The *in vivo* specific lysis indicated again that of the three tested vaccines, the most immunogenic was mMVA.HIA.85A (Fig. 3 D). For the ICS and *in vivo* killing assays, there was no statistically significant difference between the frequencies of H-specific T cells induced by the mH5 and ssp promoters. Taken together, data from three different T cell assays showed a consistent trend that the highest H-specific T cell responses were induced by the mMVA.HIVA.85A vaccine despite the fact that it did not support the highest HIVA protein expression.

### BCG.HIVA^222^-mMVA.HIVA.85A elicits oligofunctional anti-HIV-1 and anti-TB responses

**Figure 4:**
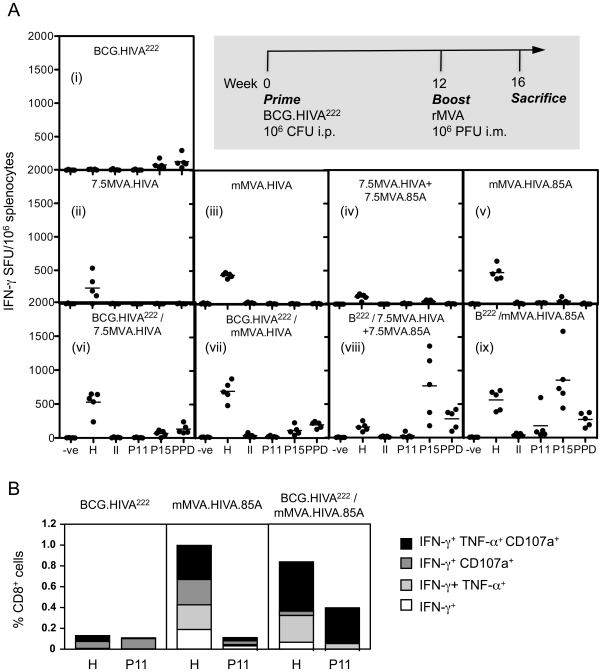
Induction of robust HIV-1- and *M. tuberculosis*-specific T cells by the BCG.HIVA^222^ prime - mMVA.HIVA.85A boost regimen. (**A**) Groups of 5 BALB/c mice received either BCG.HIVA^222^ (B^222^) (**i** and **vii–ix**) or no vaccine (**ii–v**) on week 0, followed by rMVA(s) (**ii–ix**) as indicated on the top of the graphs or no vaccine (**i**) at week 12. On week 16, mice were killed and their HIV-1 (H and II)- and mycobacterium (P11, P15 or PPD)-specific responses were determined in an IFN-γ ELISPOT assay using MHC class I (H and P11) or class II (II and P15)-restricted peptides. Results are shown as individual animal responses (black dots) with group means (horizontal bars). (**B**) The functionality of vaccine-induced CD8^+^ T cell responses was assess in a multicolour intracellular cytokine staining assay. The group mean frequencies of single-, double- or triple-cytokine-producing H- or P11-specific cells following background subtraction are shown for the three regimens indicated above the graphs.

The mMVA.HIVA.85A vaccine was tested for T cell immunogenicity in a combined regimen with BCG.HIVA^222^. Previously, we demonstrated induction of robust HIV-1-specific T-cell responses in a heterologous prime-boost regimen, in which BALB/c mice were immunized with BCG.HIVA^222^ derived from a lysine auxotroph Pasteur strain of BCG followed by a boost with 7.5MVA.HIVA [8]. However, this vaccine combination was able to benefit from a prime-boost effect only for the anti-HIVA T-cells, but could not increase responses against *M. tuberculosis*. Here, the induction of anti-Ag85A responses by the BCG.HIVA^222^ prime-mMVA.HIVA.85A boost regimen was assessed. Thus, BALB/c mice were immunized with 10^6^ CFU of BCG.HIVA^222^ i.p. or left unimmunized, and boosted with 10^6^ PFU i.m. of single or mixed rMVAs 12 weeks later or left unboosted. To control for the possible increased MVA adjuvantation given in the 7.5MVA.HIVA and 7.5MVA.85A group, additional 10^6^ PFU of empty parental MVA was added to the single rMVA immunizations to make the total number of poxvirus PFUs given to each mouse comparable. MHC class I and II-restricted T cell responses induced by immunogens HIVA and Ag85A were analyzed 2 weeks after the last vaccination and a number of observations was made. Thus, BCG.HIVA^222^ alone induced weak CD8^+^ T cell responses to P15 and weak responses to Purified Protein Derivative (PPD), the latter mainly generated by CD4^+^ T cells (Fig. 4). The P15-specific responses were strongly boosted by both the 7.5MVA.85A and mMVA.HIVA.85A vaccines (Fig. 4 A, Viii and ix). However, the lower 10^6^-PFU dose of 7.5MVA.85A and mMVA.HIVA.85A alone did not prime any significant Ag85A-specific T cells (Fig. 4 A, iv and v). Interestingly, there was a trend of augmenting the PPD responses by the prime-boost regimens (Fig. 4 A, viii and ix), while frequencies of P11-specific T cells remained low. Consistent with previous reports [25], [26], no detectable HIVA-specific T cell responses were elicited by BCG.HIVA^222^ alone (Fig. 4 A, i). A single injection of 7.5MVA.HIVA induced a typical 100- to 300-SFU response reflecting the fact that rMVA is not a strong priming vaccine (Fig. 4 A, ii and iv). This frequency was approximately doubled by using the mH5 promoter (Fig. 4 A, iii and v). A similar 2-fold increase in H-specific responses was achieved by BCG.HIVA^222^ priming of the 7.5MVA.HIVA alone responses, but not for the combination 7.5MVA.HIVA+7.5MVA85A boost (Fig. 4 A, vi and viii). A smaller BCG.HIVA^222^ priming effect was detected for the mH5 promoter-driven HIVA expression (Fig. 4 A, vii and ix). No regimen induced detectable HIVA-specific MHC class II-restricted responses. Finally, robust T cell responses against both HIVA and Ag85A antigens were elicited by the BCG.HIVA^222^-mMVA.HIVA.85A regimen (Fig. 4 A, ix), an important initial milestone for the pre-clinical development of this approach. Oligofunctionality of the vaccine-induced CD8 T cell responses was confirmed by a multicolour flow cytometry assessing production of IFN-γ, TNF-α and CD107a (Fig. 4 B).

## Discussion

In this report, we describe construction of vaccine MVA.HIVA.85A, which expresses immunogens derived from HIV-1 and *M. tuberculosis*, and demonstrate its dual T cell immunogenicity in BALB/c mice following a prime with BCG.HIVA^222^, recombinant BCG expressing the same HIV-1 immunogen. A self-excising GFP gene was used to construct a markerless MVA-vectored vaccine making it more suitable for Good Manufacturing Practice (GMP) production. Overall, we demonstrate that BCG.HIVA^222^ prime-MVA.HIVA.85A boost strategy may offer a neonate vaccination platform combining induction of T cell responses against two major killers, AIDS and TB, into a single regimen. This is an early proof-of-concept study indicating that this approach is worthy of further pursuit.

In recent years, MVA has been used extensively as a non-replicating vaccine vector for delivery of immunogens derived from diverse pathogens [27]. Its ability to boost consistently and strongly T cell responses against the transgene products in humans has been shown by experimental vaccines against AIDS, tuberculosis, malaria and cancer [10], [28]–[30]. MVA has a complex genome and its promoters are classified based on the timing of gene expression into early, intermediate and late. This has an impact on the immunogenicity through multiple mechanisms, which interfere with presentation of late T-cell epitopes [31]–[37]. Here, we compared three conventional early/late promoters P7.5, mH5 and ssp in combination with the HIVA and Ag85A open-reading frames for maximum T cell induction. The P7.5 promoter is active both early and late in the MVA life cycle and by immunofluorescence often yields low to undetectable levels of the transgene product [38], [39], yet supported good immunogenicity in a number of clinical vaccines, especially if the T cell responses were well primed, e.g. by HIV-1 infection [10], [28], [30]. The mH5 promoter is stronger than P7.5 and has an early bias, while the ssp promoter is the strongest of the three with a bias towards late expression [21], [22], [40]. We found that ssp promoter led to the highest level of protein expression, however, very high expression may lead to genetic instability as reported previously for some transgenes [21], [40], [41]. Nevertheless, stable recombinant MVAs have been prepared expressing proteins from two ssp promoters pointing away from [42] as well as towards each other [43]. Thus, genetic stability of ssp-controled transgenes is likely to be specific for each particular combination of immunogen and/or integration sites in the MVA genome [44], [45].

Next, the efficiency of T cell induction was investigated in the BALB/c mice and showed that vaccination with either mixed single-transgene or dual vaccines elicited comparable T cell responses specific for the HIVA and Ag85A proteins. Furthermore, a direct comparison of the P7.5, mH5 and ssp rMVAs showed a consistent trend in three different functional tests, the IFN-γ/CD107a ICS, IFN-γ ELISPOT and *in vivo* killing assays, that superior frequency and functionality of specific T cells against HIVA were induced by the mMVA.HIVA.85A vaccine. This outcome concurs with previously published work [22]. When the novel rMVA vaccine was tested in a heterologous BCG.HIVA^222^ prime-mMVA.HIVA.85A regimen, elicitation of robust HIV-1-specific CD8^+^ and TB-specific CD4^+^ T cell responses were detected. Although the anti-HIV-1 responses showed only a trend of enhancement by the combined regimen relative to unprimed mMVA.HIVA.85A, a boost was readily detected for the Ag85A response. HIVA-specific enhancement was observed in mice for single-immunogen as described for 7.5MVA.HIVA before [8]; this may be due to the weaker vaccinia virus promoter, which “leaves room” for improvement in this model. Overall, we find induction of the dual HIV-1 and TB-specific responses encouraging for further vaccine and regimen optimizations. Several studies used successfully rBCG in heterologous prime-boost regimens with other vaccine modalities, whereby poxviruses, adenoviruses and virus-like particles as vectors delivered a strong boost for BCG-primed responses against the shared transgene products [3], [20], [46], [47]. MVA.85A delivered a particularly strong boost for BCG-primed anti-Ag85A T cells [15], [28], [39] and in mice, intranasally administered MVA.85A increased protective efficacy of BCG against *M. tuberculosis* challenge [39]. Improved efficacy by MVA.85A over BCG vaccine alone was also shown in non-human primates and cattle [48], [49]. To prepare a BCG.HIVA vaccine compliant with Good Laboratory Practice, endosomal escape strain of BCG AERAS-401 derived from the Danish SSI-1331 parent was utilized [20]. In combination with MVA.HIVA and ovine atadenovirus-vectored OAdV.HIVA [50] vaccines, BCG.HIVA^401^ primed for robust HIV-1-specific T cell responses [20]. However, in macaque neonates, the BCG.HIVA^401^ prime-MVA.HIVA boost regimen was only weakly immunogenic [51]. It is worth noting that interspecies differences in T cell responsiveness to BCG may influence the outcome of immunizations and therefore it is not clear how adult mouse data transfer to non-human primate and human neonates; it remains a possibility that human neonates actually respond better to a rBCG vaccine than the model systems.

BCG given to immunocompromized individuals may cause a disseminated disease. Thus, the current WHO guidelines recommend withholding BCG vaccination until the HIV-1 negativity is confirmed (if the infrastructure to carry out testing exists) [52]. However, because the risk of TB is so high, HIV-1-negative babies born to HIV-1-positive mothers are recommended to receive BCG and so there would be use for BCG.HIVA. Furthermore, the lysine auxotroph strain of BCG Pasteur used here [53] may provide addition safety and genetic stability [54].

In conclusion, MVA continues to feature prominently in clinical trials of recombinant vaccines against major global diseases. Here, we have constructed a novel prototype vaccine MVA.HIVA.85A, which is markerless and therefore compatible with GMP manufacture, and combines stimulation of oligofunctional T cell responses against both HIV-1 and *M. tuberculosis*. Furthermore, we demonstrate that a dual platform against AIDS and TB consisting of BCG.HIVA prime-MVA.HIVA.85A boost is in principle possible, although particularly the HIV-1 immunogen may be further refined e.g. by using conserved regions of the HIV-1 proteome, which may better control diverse HIV-1 strains circulating in the target population and HIV-1 escape from immune responses [55]. This work is a logical extension of the current efforts in developing strategies against these two major killers and timely given the WHO's interest in improving the currently recommended infant vaccine schedules. Of course, the only relevant proof of efficacy can only come from protection studies in human neonates.

## Materials and Methods

### Preparation of dual-insert recombinant MVAs

The construction of transfer plasmids directing the insertion of transgenes under the control of either the mH5 or ssp promoters [21], [22] into the thymidine kinase (tk) locus of the MVA genome is described in detail in elsewhere generating mMVA.HIVA.85A and sMVA.HIVA.85A, respectively (DPhil Thesis, Richard Hopkins, University of Oxford, 2010). rMVAs were rescued by infecting a semi-confluent monolayer of chicken embryo fibroblast (CEF) cells grown in Dulbeco's Modified Eagles Medium supplemented with 10% FBS, penicillin/streptomycin and glutamine, (DMEM-10) with parental MVA expressing the red fluorescent protein (RFP) at multiplicity of infection (MOI) of 0.1 and transfecting 2 µg of transfer plasmid DNA containing the GFP gene as a marker 1 h later. Note, that a successful recombination into the tk locus exchanges the parental RFP for GFP of the recombinant (Gilbert, unpublished). Forty-eight h post transfection, the total virus was harvested and used to re-infect fresh CEF monolayer. rMVA was subjected to at least five rounds of plaque purification, based on GFP expression and the absence of RFP. Removal of the parental MVA was confirmed by PCR. Master virus stock was grown, purified on a 36% sucrose cushion, titred and stored at −80°C until use. In the mMVA.HIVA.85A, the GFP gene flanked by two small regions of homology, which allowed GFP removal in a homologous recombination event during further passaging and generation of a markerless vaccine (Fig. 1).

### Preparation of BCG.HIVA^222^ vaccine

Construction and preparation of the BCG.HIVA^222^ stock was described previously [26]. Briefly, mycobacterial cultures were grown in Middlebrook 7H9 broth medium or on Middlebrook agar 7H10 medium supplemented with albumin-dextrose complex (ADC, Difco) and containing 0.05% Tween 80 and 25 µg/ml kanamycin. The L-lysine monohydrochloride (Sigma) was dissolved in distilled water and used at a concentration of 40 µg/ml.

### Quantitation of HIVA and Ag85A protein expression by immunofluorescence and flow cytometry

Three hundred thousand CEF cells were infected with rMVA at 1 MOI in a single well of a six-well plate with or without a glass slide. After 16 h, cells not growing on a glass slide were detached and transferred into to a test tube. Both suspended and glass-attached CEF populations were fixed, permeabilized with −20°C methanol for 5 min, washed 3× with PBS, blocked for 30 min in PBS-10% FCS at room temperature, incubated with primary anti-HIV-1 Gag p24 antibody (NIH AIDS Reagents) at 1 µg/ml at room temperature for 2 h and washed with PBS 3×. The secondary antibody, Alexa-Fluor 594-conjugated goat anti-mouse mAb (Molecular Probes, Invitrogen), was then added at 5 µg/ml in combination with a primary anti-Pk antibody conjugated to FITC (Abcam) in PBS-1% FCS at room temperature for 2 h, and the cells were washed 3× with PBS. Cells mounted on a glass slide were covered with Vector-Shield containing DAPI (Vector Laboratories) and photographed on a Zeiss fluorescence microscope, while cells in suspension were acquired on a Cyan FACS machine (Dako) and analyzed using Flowjo software (Tree Star).

### Mouse immunizations and isolation of splenocytes

Groups of 5- to 6-week-old female BALB/c mice were immunized either without anaesthesia intraperitoneally (i.p.) with BCG.HIVA^222^, or under general anaesthesia intramuscularly (i.m) with rMVA at doses and schedules outlined in the figure legends. On the day of sacrifice, individual spleens were collected and splenocytes were isolated by pressing spleens through a cell strainer (Falcon) using a 5-ml syringe rubber plunger. Following the removal of red blood cells with RBC Lysis Buffer (Sigma), the splenocytes were washed and resuspended in RPMI 1640 supplemented with 10% FCS, penicillin/streptomycin (R-10).

### Ethics statement

All animal procedures and care conformed strictly to the United Kingdom Home Office Guidelines under The Animals (Scientific Procedures) Act 1986. The protocol was approved by the local Research Ethics Committee (Clinical Medicine, University of Oxford). Experiments were carried out under Project Licence no. 30/2406 held by TH with a strict implementation of the Replacement, Reduction and Refinement (3Rs) principles.

### Peptides

Peptides RGPGRAFVTI designated H [56] and derived from HIVA, and EWYDQSLGSVVMPVGGQSSF designated P11 and derived from Ag85A [57] were used to investigate the MHC class I-restricted responses. A pool of three peptides MHQALSPRTLNAQVKVIEEK, NPPIPVGDIYKRWIILGLNK, FRDYVDRFFKTLREAQATQE designated II [8] and a single peptide TFLTSELTGWLQANRHVKPT designated P15 [57] were used to analyze the MHC class II-restricted responses induced by immunogens HIVA and Ag85A, respectively. All peptides were synthesized in an in-house facility (Weatherall Institute of Molecular Medicine, Oxford, UK), dissolved in DMSO (Sigma) at a concentration of 10 mg/ml, and stored at −80°C. The final assay concentration for individual peptides was 4 µg/ml unless otherwise stated. PPD (Statens Serum Institute, Copenhagen) was used to assess the immunogenicity of BCG.

### *Ex vivo* IFN-γ ELISPOT assay

The ELISPOT assay was performed using the IFN-γ ELISPOT kit (Mabtech) as described previously [58]. The ELISPOT plate membranes (Millipore) were coated with purified anti-mouse IFN-γ antibody diluted in carbonate-bicarbonate buffer (Sigma) to a final concentration of 5 µg/ml at 4°C overnight, washed once in R-10, and blocked for 2 h with R-10. A total of 2.5×10^5^ splenocytes were added to each well, stimulated with HIVA- or Ag85A-derived peptides or PPD RT49 (Statens Serum Institute, Denmark) or left un-stimulated.for 16 h at 37°C, 5% CO_2_ and lyzed by incubating 2× with deionized water for 5 min. Wells were then washed 3× with PBS 0.05% Tween-20, incubated for 2 h with a biotinylated anti-IFN-γ antibody diluted in PBS 2% FCS to a final concentration of 2 µg/ml, washed 3× in PBS 0.005% Tween-20 and incubated with 50 mg/ml horseradish peroxidase-conjugated to avidin in PBS 2% FCS. Wells were washed 4× with PBS 0.005% Tween-20 and 2× with PBS before incubating with an AEC substrate solution [3-amino-9-ethyl-carbazole (Sigma) dissolved at 10 mg/ml in Dimethyl formaldehyde and diluted to 0.333 mg/ml in 0.1 M acetate solution (148 ml 0.2 M acetic acid and 352 ml 0.2 M sodium acetate in 1 liter pH 5.0) with 0.005% H_2_O_2_]. After 5–10 min, the plates were washed with tap water, dried and the resulting spots were counted using an ELISPOT reader (Autoimmune Diagnostika GmbH). All samples were analyzed in duplicates.

### Polychromatic flow cytometry assay

Two million splenocytes per well of a 96-well round-bottomed plate (Falcon) were pulsed with peptides together with anti-CD107a/b-FITC antibody and incubated at 37°C, 5% CO_2_ for 90 min before addition of GolgiStop. After further 5 h, the reaction was terminated, the cells were washed with FACS wash buffer (PBS, 2% FCS, 0.01% Azide) and blocked with anti-CD16/32 at 4°C for 30 min. All subsequent antibody incubations were performed using the same conditions. The cells were washed 2× and stained with: 25 ng of anti-mouse CD19-Pacific Blue; 100 ng anti mouse-CD3 PerCP-Cy5.5 (eBioscience); 200 ng anti-mouse CD8α-PE-Texas Red (Abcam). The cells were washed 2× with FACS buffer and permeabilized with BD Cytofix/Cytoperm (BD Biosciences), washed 2× with BD Perm/Wash buffer, before staining with 25 ng of anti-mouse IFN-γ -PE-Cy-7. Cells were washed with Perm/Wash buffer and fixed with CellFIX and stored at 4°C until analysis. Samples were acquired on Cyan FACS machine (Dako) and the results were analyzed using Flowjo software (Tree Star).

### *In vivo* killing assay

To prepare the targets, naïve 5- to 6-week-old female BALB/c mice were sacrificed and the splenocytes were isolated as described above [26]. The splenocytes were then incubated without or with 0.1, 1 or 10 µM peptide H in R-10, washed 3× and subsequently labeled with carboxyfluorescein diacetate succinimidyl ester (CFSE, Molecular Probes) at the following concentrations for the deferentially pulsed populations: 4 nM - no peptide; 16 nM–0.1 µM peptide H; 80 nM–1 µM peptide H; 160 nM–10 µM peptide for at 37°C for 15 min and a further 15 min in fresh medium. Peptide-pulsed splenocytes were washed 3× and combined for intravenous adoptive transfer. Each animal received approximately 2×10^6^ cells of each population and was sacrificed 5 h later, and its splenocytes were isolated and analyzed using flow cytometry. Cytotoxicity was calculated as described previously [59]: Adjusted % survival = 100×(% survival of peptide-pulsed targets/mean % survival of irrelevant peptide pulsed cells), followed by the calculation of % specific lysis = 100 – adjusted % survival.

### Statistical analysis

Statistical significance was determined using an unpaired Student's t-test with a two-tailed distribution on group immunization data using Prism software. Data were presented as mean ± SD unless otherwise stated. Differences were considered as significant at p≤0.05.
